# The Balance of Beauty: Pooled Analysis of Adverse Events With a Cohesive Polydensified Matrix-Hyaluronic Acid Filler in Nasolabial Fold Treatments

**DOI:** 10.1093/asjof/ojaf151

**Published:** 2025-11-27

**Authors:** Sonja Sattler, Korin Leffler, Matthias Hofmann, Ivanti Galloway, Mimi R Borrelli

## Abstract

**Background:**

Hyaluronic acid (HA) fillers are among the most widely used aesthetic treatments, valued for their safety, biocompatibility, and reversibility. Product-specific adverse events (AEs) are important to understand, especially delayed AEs.

**Objectives:**

To evaluate the incidence, severity, and risk factors of AEs following nasolabial fold (NLF) treatment with CPM-B (cohesive polydensified matrix hyaluronic acid filler with 22.5 mg/ml HA; Belotero Balance®; Anteis S.A., Switzerland) across 5 internal clinical studies.

**Methods:**

Pooled data included treatments to 526 NLFs in 412 patients. Adverse events were categorized into 12 subtypes and rated as mild, moderate, or severe. Multivariable logistic regression was used to evaluate the independent association of patient (age, sex) and procedure-related factors (injection volume, NLF laterality) with AE risk (≥1 vs 0).

**Results:**

Adverse events were observed in 41% of treated NLFs; the majority (97%) were mild, with no severe AEs documented. The most common AEs included swelling (23%), erythema (20%), and bruising/ecchymosis (17%). Adverse event risk significantly increased with injection volume; every additional 100 µl raised the odds of an AE by 2.38 (*P* = .00537). Patient age, sex, and NLF laterality were not significantly associated with AE risk.

**Conclusions:**

This study supports the overall safety and tolerability of CPM-B, with most AEs being mild and transient; only 3.8% were moderate, and none were severe. When controlling for study, volume was the only significant predictor of AE risk, highlighting the need for physicians to minimize excessive volumes where possible. Future studies should explore AE predictors in more diverse populations and investigate additional risk factors to optimize safety and outcomes.

**Level of Evidence: 3 (Risk):**

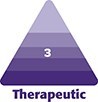

After neurotoxins, hyaluronic acid (HA) treatments are the most common non-surgical procedure worldwide.^1^ Over the past 2 decades since their introduction, the demand for HA fillers (and non-invasive cosmetic procedures generally) has risen substantially alongside the normalization of aesthetic treatments by media/celebrity influencers, the ageing population, and the general public's increased focus on health.^2-4^ The safety profile of HA fillers has contributed significantly to their popularity—HA fillers are non-immune reactive, biocompatible, and well-tolerated.^5,6^ The key ingredient within these fillers, HA, is a natural glycosaminoglycan abundant in the extracellular matrix (ECM) and has a structure highly evolutionarily conserved between species—meaning HA sourced from non-human (avian or bacterial) sources for filler manufacturing is identical to native human HA.^7^ This also means filler HA is enzymatically degraded within the body like endogenous HA making HA filler treatments reversible with synthetic hyaluronidase.^8-10^

Although HA filler treatments are generally safe, adverse events (AEs) are an inherent risk with any clinical intervention. The absolute number of AEs following HA treatments has risen with increased treatment uptake.^11^ Post-market safety surveillance is essential to monitor long-term safety and preventively mitigate AE risk.^12, 13^ AEs following HA fillers are classified as acute or delayed based on time-of-onset;^14^ typically, those occurring within 14-days and resolved by 7 days are acute (early-onset),^2^ and any AE beyond 4-weeks is delayed.^14^ The majority of AEs following HA fillers are acute, mild, and transient events (eg, local edema, erythema, bruising) that can be minimized with appropriate procedural technique.^1, 2, 6, 7^ Delayed AEs, in contrast, are more concerning since they tend to be more extreme and longer lasting.^15^ Delayed AEs are commonly caused by patient-specific immune reactions to specific HA products (eg, hypersensitive reactions, granulomas, and nodules),^2, 16, 17^ and are therefore minimized with appropriate product selection.

Adverse events are estimated to occur in 33.3% of HA filler treatments, but incidence ranges from 2% to 74% depending on the specific HA filler implanted.^2^ Because delayed AEs are often product-specific, product selection and knowledge of product-specific AEs is critical, but data on this topic are lacking.^2^ This manuscript therefore focused on AEs following treatments using the cohesive polydensified matrix HA (CPM-HA) collection (Belotero®, Anteis S.A Plan-les-Ouates, Switzerland, a company of the Merz Aesthetics® group).

The CPM-HA fillers are manufactured using a patented cohesive polydensified matrix (CPM) technology, which creates HA gels which exhibit polydensity in crosslinking density throughout their substance. This unique feature enhances gel cohesivity and facilitates tissue penetration.^18-21^ The microscopic appearance of the CPM-HA-treated dermis more closely resembles the native human dermis than does a dermis treated with non-CPM-HA fillers,^21-24^ which has led to description of the CPM-HA fillers as “biomimetic.” The first CPM gel on the global market was CPM-B (Belotero® Basic/Balance with 22.5 mg/ml of HA)—launched in Germany in 2005 and approved for injections into the mid-to-deep dermis for correction of moderate-to-severe facial wrinkles and folds by the Food and Drug Administration (FDA) in 2011.^25^ Since these introductions, CPM-B has established an excellent safety profile.^26, 27^ It remains one of the few dermal fillers able to be injected superficially (via the blanching technique) into high-risk facial areas without causing a Tyndall effect.^28^

To our knowledge no systematic review or meta-analysis has evaluated AEs specific to CPM-B. This analysis therefore pooled data reporting on AEs following CPM-B treatments to the nasolabial folds (NLFs) from 5 internal post marketing studies (Merz Pharmaceuticals, GmbH & Co. KGaA, Frankfurt, Germany). Adverse event prevalence, type, and severity were analyzed, and AE risk factors were investigated in this large patient cohort.

## METHODS

Data were extracted from the 5 internal pivotal and post-marketing studies (Merz Pharmaceuticals GmbH, Frankfurt am Main, Germany) reporting on clinical outcomes and AEs following treatment with CPM-B to the NLF. All reports were finalized between 2002 and 2012 (Oxford Level of Evidence: II^29^). Primary endpoints varied, but all studies reported data on similar categories of AEs enabling pooling (Table 1).

**Table 1.** Details of the 5 Internal Pivotal and Post-Marketing RCTs Reporting on AEs Following CPM-B Treatment to the NLF/s

	Study	1	2	3	4	5
Study Details	Location	Frankfurt, Germany	Frankfurt, Germany	Hamburg, Germany.	Hamburg, Germany	Beijing, China.
	Design	Prospective, randomized, subject- and rater-blinded split-face, single center.	Prospective, multicenter, non-controlled study, four centers.	Prospective, rater-blind, randomized, split-face trial, single center.	Prospective, randomized, split-face, single-center.	Prospective, randomized, double blind, controlled, split face, multicenter.
	Phase	IV	IV	IV	IV	IV
	Follow-up	4-weeks	36-weeks (24-week main phase, optional 12-week follow-up).	4-weeks	4-weeks	52-weeks
	Patients	40	114	20	20	220
	NLFs	40	228	40	40	440
	Level of evidence	II	II	II	II	II
AE	Classification	Mild, moderate, severe.	Mild, moderate, severe.	Mild, moderate, severe.	Mild, moderate, severe.	Mild, moderate, severe.
	Assessment	Physical exam (week 0, week 2, week 4).	Physical exam at follow-up.	Physical exam (at baseline, treatment, weekly to 4-weeks).	Physical exam at (at day of treatment, weekly to 4-weeks).	Physical exam at (at day of treatment, weekly to 4-weeks).
	Outcome	Mild AEs, similar between treatments; no severe AEs recorded.	Most AEs were mild and resolved without intervention; no severe AEs recorded.	All adverse events mild; no significant differences between treatments in safety profile.	Mild pain differences between fillers; no severe AE.	All related AEs were mild.

AEs, adverse outcomes; GCP, good clinical practice; MedDRA, Medical Dictionary for Drug Regulatory Activities Terminology; NLF, nasolabial fold.

All AEs per NLF were recorded without limitation on the total number of AEs per NLF. For split-face studies, only the side treated with CPM-B was analyzed. In cases where both NLFs were treated but AE laterality and/or volume injected was not specified, the total number of AEs and/or volume injected for that patient was evenly divided between both NLFs (this meant AEs could increase in fractional increments, and since all AEs were rounded up, the total AEs recorded per patient were conservatively inflated resulting in a discrepancy between Supplemental Tables 1 and 2). The categorization of AE severity (mild, moderate, or severe) at each NFL was also retrieved.

Variables considered in this analysis included study number (1, 2, 3, 4, or 5), patient age in years, patient sex, patient race, NLF laterality (left, right), and filler volume injected in milliliters (counted at the NLF level). In each study, the injected filler volume was at the discretion of the investigator.

### Statistics

Data were compiled in R version 4.4.1. Descriptive statistics were used to summarize the data, with continuous variables presented as mean ± standard deviation (SD) or median (range) and categorical variables as counts, frequencies, and percentages. A binary dependent variable indicating the presence of AEs (0 vs ≥1 AEs) was calculated for each NLF. Comparisons between NLF cohorts experiencing 0 vs ≥1 AEs were performed using 2-sided T-tests for continuous variables and Pearson's chi-squared test or Fisher's exact test for categorical variables. Injection volume was analyzed as a categorical variable using Pearson's chi-squared test with simulated *P*-values (2000 replicates) to assess its association with AE occurrence. This approach was chosen due to the non-parametric, discrete distribution of volume data.

Logistic regression analysis was employed to model the association between various factors and the likelihood (log-odds) of experiencing an AE post-procedure. The independent variables within the model included age, volume, laterality, sex, and race. The study indicator variable was included in the model to account for heterogeneity among the studies (eg, date, location, injector). A multivariable logistic regression analysis was then performed, including all independent variables, to assess their individual associations. Results were reported as odds ratios (ORs) and 95% confidence intervals (CIs). Statistical significance was determined at a *P*-value threshold of <.05.

## RESULTS

### Demographics

The data pooled across all 5 clinical studies consisted of 526 NLF sites in 412 individual participants (aged 22-79 years); 114 participants contributed both NLFs and 298 contributed either the left or right NLF. The mean age (±SD) was 47 ± 9.4 years, 47% of all participants (*n* = 193/412) were white, and the remaining participants (53%, *n* = 218/412) were all from Study 5 (conducted in China) and thus were presumed Asian. The total number of patients included in Study 5 was 220; however, since volume was not recorded for 2 participants, data were only included from the 218 participants from Study 5 who had complete data. Most participants were females (96%, *n* = 394/412). Treatments were administered to 263 (50%) right and 263 (50%) left NLFs. The average volume of CPM-B injected was 1.15 ± 0.38 mL (Table 2).

**Table 2.** Variables Across the 5 Clinical Study Reports

Study		1	2	3	4	5	Total
Year published		2006	2010	2011	2012	2002	2006-2012
Total patients, *n*		40	114	20	20	218	412
Total NLF sites, *n*		40	228	20	20	218	526
NLF	Right, *n* (%)	21 (52.5)	114 (50)	10 (50)	10 (50)	108 (49.5)	263 (50)
	Left, *n* (%)	19 (47.5)	114 (50)	10 (50)	10 (50)	110 (50.4)	263 (50)
Age	Mean ± SD	52.6 ± 8.73	50.23 ± 7.09	45.8 ± 7.14	45.9 ± 6.66	43.56 ± 9.83	46.5 ± 9.4
	Median (Range)	53 (33-69)	52 (30-60)	44.5 (34-59)	46.5 (35-64)	43 (22-73)	47 (22-73)
Sex	Female, *n* (%)	36 (90)	103 (90.35)	19 (95)	19 (95)	209 (96)	386 (93.7)
	Male, *n* (%)	4 (10)	11 (9.65)	1 (5)	1 (5)	9 (4)	26 (6.3)
Race	White, *n* (%)	40 (100)	114 (100)	20 (100)	20 (100)	0 (0)	194 (47)
	Asian, *n* (%)	0 (0)	0 (0)	0 (0)	0 (0)	218 (100)	218 (53)
Volume (/1 mL)	Mean ± SD	1.43 ± 0.37	0.99 ± 0.24	1.41 ± 0.47	1.42 ± 0.40	1.22 ± 0.4	1.15 ± 0.38
	Median (range)	1.4 (0.65-2)	1 (0.45-1.5)	1.15 (1-2)	1.35 (1-2)	1.2(0.3 -2)	1.13 (0.3-2)
AE	≥1, *n* (%)	26 (65)	140 (61.4)	8 (40)	10 (50)	34 (15.6)	218 (41)
	0, *n* (%)	14 (35)	88 (38.6)	12 (60)	10 (50)	184 (84.4)	308 (59)
Total AEs/participant	Mean ± SD	1.13 ± 0.99	0.85 ± 0.94	0.5 ± 0.69	0.6 ± 0.68	0.2 0.52	0.58 ± 0.76
	Median (range)	1 (0-3)	0.5 (0-6)	0 (0-2)	0.5 (0-2)	0 (0-1)	0.31 (0-6)

AE, adverse event; NLF, nasolabial fold; SD, standard deviation.

### Adverse Events

**Figure 1.**
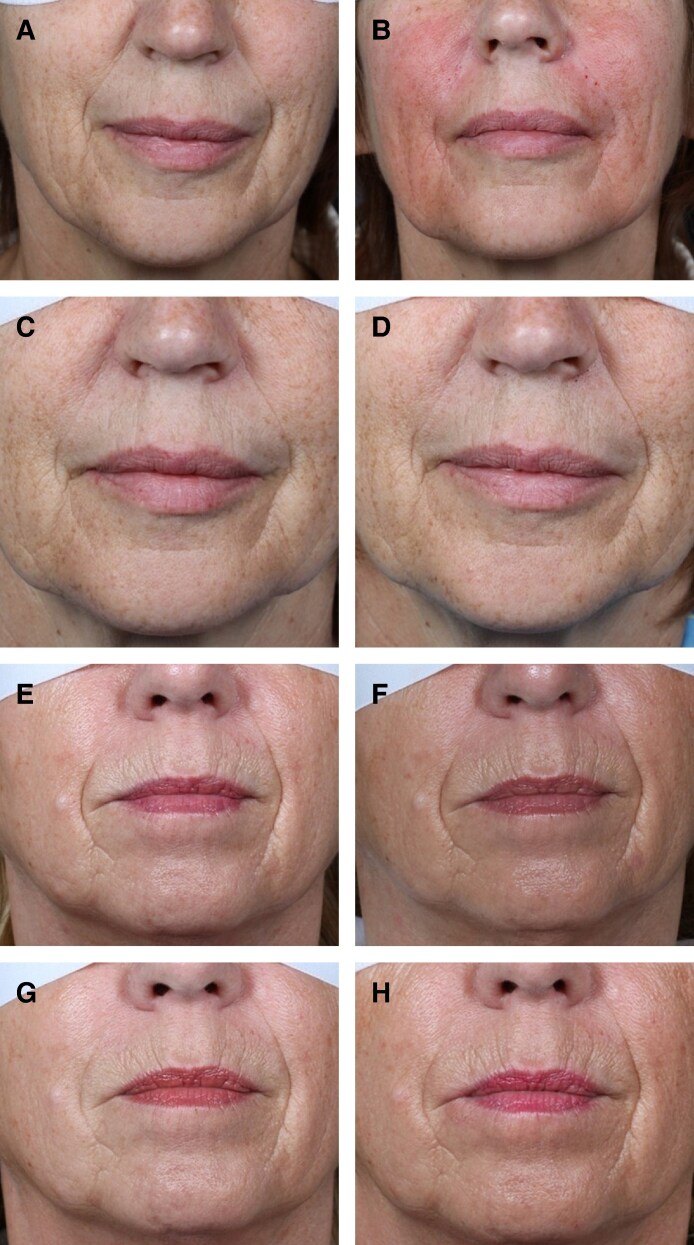
Clinical photographs of 2 female patients with documented adverse events (AEs). Patient 1 (54 years old) is shown at (A) baseline, (B) immediately after having received 1.3 mL of CPM-B to the left NLF, (C) at post-treatment Visit 3, and (D) at post-treatment and Visit 4; she experienced 3 mild AEs (swelling/pressure, bruising/ecchymosis, and erythema). Patient 2 (61 years old) is shown (E) at baseline, (F) immediately after having received 1.5 mL of CPM-B to the left NLF, (G) at post-treatment Visit 3, and (H) at post-treatment and Visit 4; she experienced 2 mild AEs (swelling/pressure and bruising/ecchymosis). AE timepoints were not recorded.

A total of 376 AEs were documented in the 526 treated NLFs. Less than half (41%, *n* = 218) of the NLFs experienced one or more AEs. The median number of AEs per participant was 0.58, with a range of 0 to 6 (Table 2). The distribution of AEs is detailed in Supplemental Table 1: 105 NLFs (19.96%) had 1 AE, 61 NLFs (23.19%) had 2 AEs, 25 NLFs (14.26%) had 3 AEs, 13 NLFs (9.89%) had 4 AEs, 2 NLFs (1.9%) had 5 AEs, and 2 NLFs (2.28%) had 6 AEs. Almost all the AEs were mild (n = 354/376, 94.15%) with the few remaining classified as moderate (*n* = 22/376, 5.85%); no severe AEs were documented (Supplemental Table 1). The most frequently reported AE was swelling (*n* = 83/374, 22.19%), followed by erythema (n = 74/374 19.79%), bruising/ecchymosis (*n* = 62/374, 16.58%), induration (*n* = 31/374, 8.29%), and pruritus (*n* = 29/374, 7.75%; Supplemental Table 2). Two representative patients documented to have ≥1 AEs are shown in Figure 1. The NLF sites with no AEs were not significantly different from NLF sites that had ≥1 AE in terms of NLF laterality (*P* = .6363), but age (*P* = .04) and volume injected (*P* = .0004) were higher in NLFs that had ≥1 AEs vs 0 AEs (Table 3). Three studies (Studies 1, 3, and 4) monitored AEs up to 4 weeks, whereas Study 2 extended monitoring up to 36-weeks, and Study 5 monitored participants up to 52 weeks post-treatment. In Study 2, 228 NLFs were treated, and 189 total AEs were documented, of these, 5 (2.95%) were nodules—and all these nodules were mild and attributed to injection-related factors. In Study 5, 218 NLFs were treated, and 32 total AEs were documented. Of these, 13 nodules (5.96%) were documented at the injection site—all were mild and only palpable not visible.

**Table 3.** Comparison of Variables Experienced at NLFs With 0 vs ≥1 AEs

Variable		0 AE (*n* = 124)	≥1 AE (*n* = 184)	*P* value
Laterality	Left (*n* = 263), *n* (%)	152 (29)	113 (21)	.6363
	Right (*n* = 263), *n* (%)	156 (30)	105 (20)	
Volume (mL)	Mean ± SD	1.15 ± 0.34	1.16 ± 0.35	.0004
	Median (range)	1 (0.3-2)	1 (0.45-2)	
Age (years)	Mean ± SD	46.21 ± 9.03	48.88 ± 8.95	.04
	Median (range)	50.5 (22-68)	51 (28-71)	

AE, adverse events; NLFs, nasolabial folds; SD, standard deviation.

All variables total *n* = 526 observations (NLFs) in 412 patients.

### Identifying Adverse Event Risk Factors

A multivariable logistic regression identified injection volume, and study as significant predictors of AEs (Table 4). Race was not included as a variable because it was confounded by study site. Higher injection volumes were strongly associated with an increased risk of AEs; every 100 µl increase in filler volume raised the odds of having an AE by approximately 2.38 times (OR: 2.38; 95% CI: 1.29-4.38; *P* = .00537). Compared to Study 1 (reference group), Study 3 (OR = 0.30; 95% CI: 0.10-0.95; *P* = .040) and Study 5 (OR = 0.09; 95% CI: 0.04-0.20; *P* < .001) were associated with significantly lower AE risk. In contrast, age, sex, and laterality were not significantly associated with AE risk. Variability between study sites also influenced AE likelihood; certain sites had a higher propensity for AEs, highlighting the importance of site-specific factors in the analysis.

**Table 4.** Multivariable Logistic Regression for Predictors of ≥1AE

Independent Variables	OR	95% CI	*P*-value
Age (years)	0.980787	0.95602-1.00620	.1370
Volume (100 µl)	3.099	1.607-5.975	.0007
Sex (ref: male)	0.471	0.205-1.082	.0759
Laterality (ref: right side)	0.849	0.572-1.261	.4180
Study Site (ref: 1) vs Study 2			
Study 3	1.33	0.62-2.85	.459
Study 4	0.30	0.10-0.95	.040
Study 5	0.46	0.15-1.41	.173

Multivariable logistic regression; dependent variable: ≥ 1AE (yes/no), controlled (categorical) variable: Study (1, 2, 3, 4, or 5, reference: Study 1).

LR chi2(3): 8.52; Prob > Chi2: .2890.

CI, confidence interval; OR, odds ratio; SE, standard error of the mean.

All variables total *n* = 526 observations (NLFs) in 412 patients.

## DISCUSSION

Hyaluronic acid (HA) filter treatments have increased over the last 2 decades since their introduction to the clinical market, which has been associated with an increase in the absolute number of adverse events (AEs) following these treatments.^2-4^ Delayed AEs are less common than acute AEs, but carry greater clinical significance due to their severity and permanence.^30^ Since delayed AEs are usually specific to the exact HA filler injected,^17,28,31-33^ knowledge of product-specific AEs can help mitigate their risk.^30^ This analysis pooled data on AEs following CPM-B treatments to 526 NLFs from 5 pivotal and post-marketing studies to investigate their prevalence, type, and severity. A regression analysis was also performed to explore which patient- and injection-factors significantly increased the risk of AEs following these CPM-HA treatments.

Adverse events were documented in 41% of cases—consistent with the reported prevalence of AEs following HA treatments generally.^16^ One prior comparative study found the incidence of AEs following CPM-B filler treatments was 1% of cases (intermittent edema only), but 2%-28% with other HA fillers.^34^ Almost all AEs in this pooled analysis were mild (94.14%), and no severe AEs were reported. The most common AE subtypes were swelling, erythema, bruising/ecchymosis, and pruritus—all considered acute, localized, transient, and attributable to injection technique.^35–38^ Study was included as a controlled factor in the regression analysis to adjust for inter-study differences (eg, location, injector experience, patient population, and follow-up duration). These differences likely explain the significantly lower likelihood of AE in Studies 3 and 5 compared to Study 1. When controlling for study, the only significant independent predictor of AE likelihood was filler volume. Filler volume has previously been linked to AE incidence,^3^ which highlights the need to exert caution with larger injection volumes, or avoiding large injection volumes where possible (for example, multiple smaller injections, even spaced across time).

Although AE time-of-onset was not reported in the included studies, the incidence of AE subtypes more commonly recognized as delayed were further explored. Induration occurred in 8.29% of treated NLFs in this analysis—whereas induration is reported to occur in 28% of all HA filler treatments.^2^ Acute, non-specific procedural reactions, poor injection technique, highly viscous fillers, and more chronic causative factors (prolonged inflammation, fibrosis or immune responses) are identified as factors causing induration.^39^ Nodules occurred in 10.46% of cases, almost half the rate reported following all HA filler treatments in a recent analysis of the recent Manufacturer and User Facility Device Experience (MAUDE) database (at 23.9%).^2^ However, this comparison is made with caution since reporting in the MAUDE database is voluntary. Nodules are typically attributed to procedure-related causes (suboptimal filler placement) or to reactions to the filler material.^2^ Numbness occurred following 0.8% of cases, similar to published rates at 1%.^2,7^ Numbness can be a symptom of nerve compression or inflammation. Discoloration occurred in 1.07% of cases. Although data on the incidence of “discoloration” following HA filler treatments collectively is lacking, and its etiologies are numerous, the rates of one specific (blue) discoloration, the Tyndall effect, have been investigated. No incidence of the Tyndall effect has been observed following CPM-HA filler treatments,^18,40–43^ but a recent systematic review reported this complication occurs in 3.17% of HA treatments collectively.^44^

This study is retrospective which introduced several limitations since the analysis was limited to only the data recorded as part of the original 5 studies. Importantly, 84.7% of the injected NLFs originated from only 2 of the 5 contributing studies, which may introduce selection bias. Furthermore, the relatively short follow-up durations in 3 of the 5 studies may have limited the detection of delayed-onset AEs, potentially skewing the overall safety profile. The included studies made no distinction between acute/delayed AEs nor between treatment-related/unrelated AEs, which limited conclusions about these AE subtypes. Direct comparisons are required to validate the observations of reduced rates of more delayed-like AEs (nodules, induration, numbness). The diversity among the study population was restricted limiting generalizability. Lastly, all studies included were internal to the product manufacturer, which may have introduced bias. Future work using prospective comparative studies including data on AE subtype in a more diverse patient cohort can help validate the results here.

Delayed AEs (adverse events) following HA filler treatments are concerning due to their severity and permanence. Since delayed AEs are often product-specific, data on AEs following specific HA filler products inform product selection to mitigate risk. Future studies should validate these findings in more diverse patient populations and explore additional factors, including injector experience and technique, to further optimize risk stratification and enhance safety in HA filler treatments.

## CONCLUSIONS

This analysis focused on AEs following CPM-B treatments to the nasolabial folds (NLFs). The AEs were mostly mild and transient. When controlling for study, the only significant predictor of AE likelihood was filler volume. These data corroborate the safety of CPM-B and highlight the need to not deliver more than the indicated filer volume.

## Supplemental Material

This article contains supplemental material located online at https://doi.org/10.1093/asjof/ojaf151.

## Supplementary Material

ojaf151_Supplementary_Data
