# Supplementary material for: The Balance of Beauty: Pooled Analysis of Adverse Events With a Cohesive Polydensified Matrix-Hyaluronic Acid Filler in Nasolabial Fold Treatments
Source: Aesthet Surg J Open Forum. 2025 Nov 27;7:ojaf151. doi: 10.1093/asjof/ojaf151 (PMC12709287; doi:10.1093/asjof/ojaf151)
Supplement: ojaf151_Supplementary_Data [file ojaf151_supplementary_data.docx]

| **Supplementary Table 1. Total number of AEs Recorded and AE Severity across all Five Studies.** | | | | | | | | |
| --- | --- | --- | --- | --- | --- | --- | --- | --- |
|  | **Study** | | | | |  | **Severity**** | |
| **AE number** | **1**  **(n=40)** | **2**  **(n=228)** | **3**  **(n=40)** | **4**  **(n=40)** | **5 (n=218)** | ***Totals AEs/pt**** | **Mild** | **Moderate** |
| 1 | 12 | 47 | 6 | 8 | 32 | *105* | 105 | 0 |
| 2 | 11 | 45 | 3 | 2 | 0 | *122* | 59 | 2 |
| 3 | 3 | 22 | 0 | 0 | 0 | *74* | 23 | 2 |
| 4 | 0 | 13 | 0 | 0 | 0 | *52* | 10 | 3 |
| 5 | 0 | 2 | 0 | 0 | 0 | *10* | 2 | 0 |
| 6 | 0 | 2 | 0 | 0 | 0 | *12* | 2 | 0 |
| **Total** | **26** | **140** | **8** | **10** | **32** | ***376*** | **354** | **22** |
| *Total AEs was 374 however the total number of AEs reported here (376) is due to conservative rounding; when bilateral NLFs were treated but AE laterality was not specified, total AEs recorded per patient were evenly divided between NLFs, and if this resulted in fractional increments, AEs were rounded up, which conservatively inflated the total AEs recorded per patient.  **AE severity was recorded per patient, not per AE, based on the most significant AE experienced. No severe AEs were observed in any of the 5 internal studies.  Abbreviations: AEs – adverse events, pt – participant. | | | | | | | | |

**Supplementary Table 2**

| **Supplementary Table 2. Adverse Events (AEs) by Category and Subcategory.** | | | |
| --- | --- | --- | --- |
| **Category** | **Subcategory** | **N** | **% of Total 374*** |
| **General** | ***Total*** | 152 | 40.64 |
|  | *Swelling* | 83 | 22.19 |
|  | *Nodule* | 38 | 10.16 |
|  | *Induration* | 31 | 8.29 |
| **Skin** | *Total* | 128 | 34.22 |
|  | *Erythema* | 74 | 19.79 |
|  | *Pruritus* | 29 | 7.75 |
|  | *Pain* | 18 | 4.81 |
|  | *Discoloration* | 4 | 1.07 |
|  | *Numbness* | 3 | 0.80 |
| **Vascular** | *Total* | 86 | 22.99 |
|  | *Bruising/ecchymosis* | 62 | 16.58 |
|  | *Bruising* | 15 | 4.01 |
|  | *Bleeding* | 9 | 2.41 |
| **Other** | *Total* | 8 | 2.14 |
|  | *Headache* | 2 | 0.53 |
|  | *Abnormal Liver Function* | 2 | 0.53 |
|  | *Fainting* | 3 | 0.80 |
|  | *Device Migration* | 1 | 0.27 |
| * n = 218 NLFs had ≥1 AE; subcategory totals exceed category totals, and category totals exceed the number of NLFs due to multiple AEs per site. Note: Systemic AEs such as fainting are considered treatment-emergent if they occur after treatment. | | | |
